# Commensal microbiome dysbiosis elicits interleukin-8 signaling to drive fibrotic skin disease

**DOI:** 10.1093/pnasnexus/pgae273

**Published:** 2024-07-30

**Authors:** Wenyu Zhang, Qili Peng, Xian Huang, Qing Huang, Zhiliang Zhang, Fuli Li, Naisheng Zheng, Binsheng Shi, Zhihong Fan, Tomasz Maj, Rui Chen

**Affiliations:** Institute of Molecular Medicine, Renji Hospital, School of Medicine, Shanghai Jiao Tong University, Pujian Road 160, Shanghai 200240, China; Department of Surgery, Division of Plastic and Reconstructive Surgery, Renji Hospital, School of Medicine, Shanghai Jiao Tong University, Pujian Road 160, Shanghai 200240, China; Institute of Molecular Medicine, Renji Hospital, School of Medicine, Shanghai Jiao Tong University, Pujian Road 160, Shanghai 200240, China; Institute of Molecular Medicine, Renji Hospital, School of Medicine, Shanghai Jiao Tong University, Pujian Road 160, Shanghai 200240, China; Department of Surgery, Division of Plastic and Reconstructive Surgery, Renji Hospital, School of Medicine, Shanghai Jiao Tong University, Pujian Road 160, Shanghai 200240, China; Department of Surgery, Division of Plastic and Reconstructive Surgery, Renji Ningbo Hangzhou Bay Hospital, School of Medicine, Shanghai Jiao Tong University, Binhai Second Road 1155, Ningbo 315600, China; Institute of Molecular Medicine, Renji Hospital, School of Medicine, Shanghai Jiao Tong University, Pujian Road 160, Shanghai 200240, China; Institute of Molecular Medicine, Renji Hospital, School of Medicine, Shanghai Jiao Tong University, Pujian Road 160, Shanghai 200240, China; Department of Laboratory Medicine, Renji Hospital, School of Medicine, Shanghai Jiao Tong University, Pujian Road 160, Shanghai 200240, China; Department of Surgery, Division of Plastic and Reconstructive Surgery, Renji Hospital, School of Medicine, Shanghai Jiao Tong University, Pujian Road 160, Shanghai 200240, China; Department of Surgery, Division of Plastic and Reconstructive Surgery, Renji Ningbo Hangzhou Bay Hospital, School of Medicine, Shanghai Jiao Tong University, Binhai Second Road 1155, Ningbo 315600, China; Institute of Molecular Medicine, Renji Hospital, School of Medicine, Shanghai Jiao Tong University, Pujian Road 160, Shanghai 200240, China; Institute of Molecular Medicine, Renji Hospital, School of Medicine, Shanghai Jiao Tong University, Pujian Road 160, Shanghai 200240, China; Department of Surgery, Division of Plastic and Reconstructive Surgery, Renji Hospital, School of Medicine, Shanghai Jiao Tong University, Pujian Road 160, Shanghai 200240, China

**Keywords:** skin fibrosis, keloid, microbiota, IL-8

## Abstract

Wound healing is an intensely studied topic involved in many relevant pathophysiological processes, including fibrosis. Despite the large interest in fibrosis, the network that is related to commensal microbiota and skin fibrosis remains mysterious. Here, we pay attention to keloid, a classical yet intractable skin fibrotic disease to establish the association between commensal microbiota to scaring tissue. Our histological data reveal the presence of microbiota in the keloids. 16S rRNA sequencing characterizes microbial composition and divergence between the pathological and normal skin tissues. Moreover, the data show elevation of interleukin-8 (IL-8) in both the circulation and keloid tissue, which elicited the collagen accumulation and migratory program of dermal fibroblasts via CXCR1/2 receptor. Our research provides insights into the pathology of human fibrotic diseases, advocating commensal bacteria and IL-8 signaling as useful targets in future interventions of recurrent keloid disease.

**Significance Statement**

Fibrosis-related and nonhealing-related regenerative failures cause severe outcomes, both physiologically and psychologically. Though pivotal advances have been made of this field, many challenges and knowledge gaps remain to be addressed. Here, we pay attention to a classical skin fibrotic disease keloid, which is a mirror to other organs' fibrosis, even tumors, considering its evident tumor-like characteristics. Our findings show that a significant dysbiosis of commensal microbiota exists in intra-tissue, but not on the surface of lesions. Moreover, we revealed an elevation of microbiota-responsive cytokine interleukin-8 in the microenvironment, which originated from and promoted dermal fibroblasts, switching into migratory α-smooth muscle actin (α-SMA)-positive and contractile myofibroblasts. Overall, these findings provide insights into the pathophysiology of fibrosis and a promising avenue for translational practice.

## Introduction

Wound healing in adult human tissues often generates a fibrotic scar instead of tissue regeneration, which impairs quality of life and even causes death. Keloids are thick, raised skin scars that grow beyond the original wound area (1, 2). The scars are not deadly, but they cause chronic itching, pain, and cosmetic disfigurement, leading to a poor quality of life, anxiety, and depression (3). Surgery, corticosteroid medication, and radiation are not fully effective (2); therefore, we need to understand the root problem. The basis of keloid formation is fibroblast hyperproliferation and massive deposition of collagen types I and III (1, 2). The initiation and support of this fibroblastic pathology remain elusive. Previous research indicates that keloids are related to genetic features (4, 5), mechanical properties of the skin (6), nutrition and metabolism (7), composition of local hematopoietic compartment (8–10), and neuro-endocrine interactions (11). These different factors contribute to keloid formation, but no single aspect is the main cause.

Emerging evidences show links between the microbiota and the development of fibrosis. For example, intestinal microbiota is a key driver of inflammatory bowel disease, which develops further to intestinal fibrosis (12). Also, gut bacteria are linked with liver fibrosis in non-alcoholic fatty liver disease (13). Lung microbiota elicits interleukin-17B signaling pathway, which promotes the progression of pulmonary fibrosis (14). In a mouse model, genetic or chemical inhibition of toll-like receptor 4 (TLR4), essential sensor of bacterial products, significantly reduces fibrosis progression (15, 16). These studies suggest that the disturbance of the microbial community has an influence on the fibrosis of different organs. Therefore, it is reasonable to ask whether and how microbiota contribute to abnormal fibrosis of the skin.

Skin is one of the critical barrier tissues directly interacting with microbiota. The presence of microorganisms was well studied in dermal infections for decades. Recent sequencing methods show that skin is a complex ecological network of microorganisms and the host (17). It is also well established that both commensal and pathogenic microbiota affect the outcome of wound healing (17, 18). It was shown that in the systemic scleroderma skin, which is also accompanied by fibrosis, patients show microbial dysbiosis compared to normal skin samples (19). However, to our knowledge, the microbiota was not studied in keloids. In our paper, we used 16S rRNA gene sequencing of hospitalized patients to gain a landscape of the microbial community. We show dysbiosis of cutaneous microbiome between keloid lesion and non-keloid skin. Next, we analyzed potential cytokines based on single-cell RNA sequencing (RNA-Seq). We showed that interleukin-8 (IL-8), also known as CXCL8, is elevated in keloids and patients' circulation. We also checked the spatial distribution of IL-8 in the keloid microenvironment. Next, we show that IL-8 could promote profibrotic events, including migration, collagen deposition, and contraction of dermal fibroblasts. Since the bacterial community and IL-8 expression in keloids were not previously studied, the findings provide new insights into the cause of keloid and identify signals helping the proper tissue repair. Meanwhile, it will help to develop translational strategies to optimize clinical management of disease.

## Results

### Microbiota are present in keloids

**Fig. 1.**
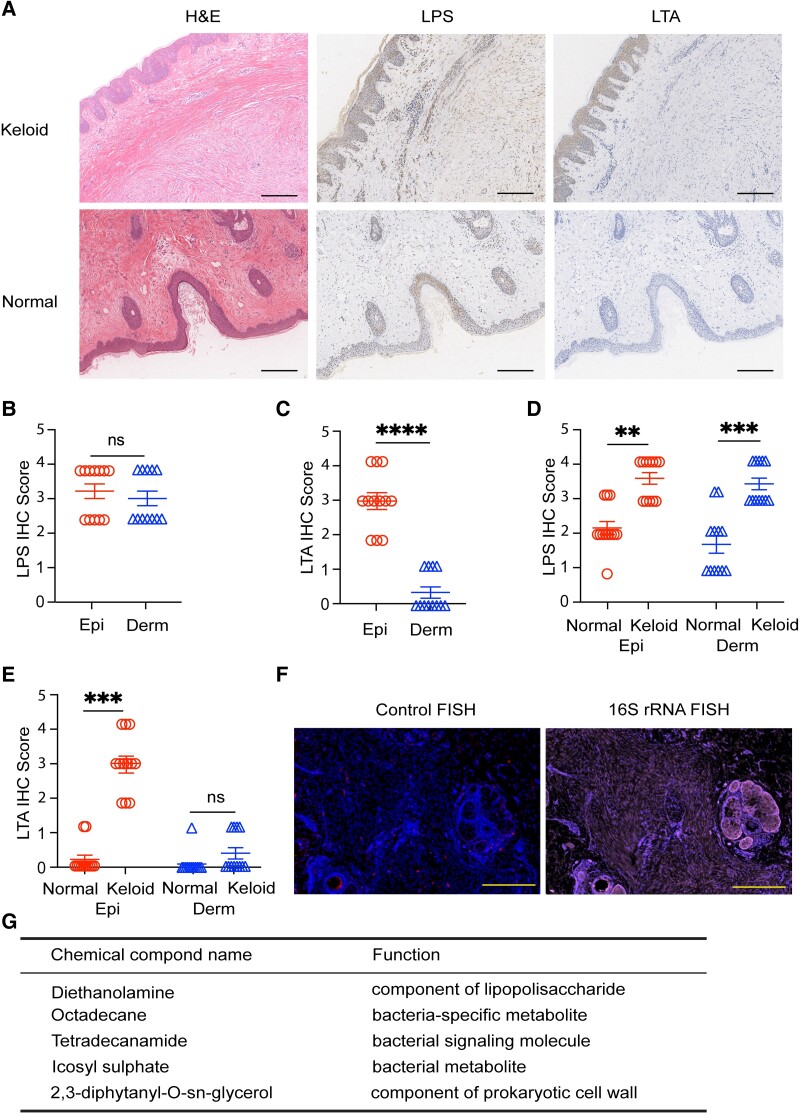
Colonization of keloid and normal skin by bacteria. A) Representative pictures of histological staining of keloid in normal tissue (upper eyelids) and IHC staining specific for LPS and LTA. Scale bars, 200 μm. Difference in IHC scoring for LPS (B) and LTA (C). Comparison of IHC scoring values between epidermis and dermis in normal and keloid skin tissues for LPS (D) and LTA (E). F) Representative picture of 16S rRNA FISH, depicting specific staining (right) and control staining with not specific oligonucleotides (left). *n* = 6; scale bars, 500 μm. G) The list of bacteria-related chemicals identified in the keloid tissues by nontargeted metabolomic analysis (*n* = 6). Statistical significance (Wilcoxon’s test): **P < 0.01; ***P < 0.001; ****P < 0.0001; ns, not significant.

To check the possibility that bacteria reside in the keloid tissues, we performed the general immunohistochemical (IHC) staining of lipoteichoic acid (LTA) and lipopolysaccharide (LPS) in clinical samples from keloids or normal upper eyelid skin. These molecules are markers of Gram-positive and Gram-negative bacteria, respectively. Both induce immune-related signaling as pathogen-associated molecular patterns (20–22). LTA was detected mainly in epidermis, but not in the deeper parts of the tissue (Fig. 1A and SI Appendix, Fig. S1A). In contrast, LPS is distributed in both epidermis and dermis (Fig. 1A and SI Appendix, Fig. S1A). These data were further characterized by detailed histological scoring. In the keloid tissue, LPS displays similar scoring values in epidermis and dermis (Fig. 1B and SI Appendix, Fig. S1B), while LTA is distributed differentially, located mainly in epidermis (Fig. 1C). Interestingly, the IHC score for LPS is higher in both epidermis and dermis of keloids compared to normal skin (Fig. 1D), while LTA shows a higher concentration in keloid than in normal epidermis, but not in dermis (Fig. 1E). It implies that microbial components in healthy and keloid skin are different.

Though IHC data suggest that keloids are colonized mainly by Gram-negative bacteria (SI Appendix, Fig. S1C), the retention of bacterial products in the tissues can last days or weeks. Therefore, they may be not representative of general bacteria configuration (23, 24). We conducted fluorescent in situ hybridization (FISH) to directly detect the bacterial 16S rRNA gene and confirm the presence of bacteria. Similar to IHC, we observed the bacterial rRNA in the keloid tissue (Fig. 1F), which was also higher compared to normal skin (SI Appendix, Fig. S1D). We also performed the nontargeted metabolomic analysis for keloid tissues (Fig. 1G). We detected microbiota-associated components, implying the presence of microorganisms. Importantly, 16S rRNA co-localizes with immunostaining of fibroblast activation protein alpha (FAP), the marker of fibroblasts in keloids (25) (SI Appendix, Fig. S1E). Together, these data show that keloid tissues contain bacteria different from normal skin.

### Keloid and healthy skin exhibit similar surface microbial community

**Fig. 2.**
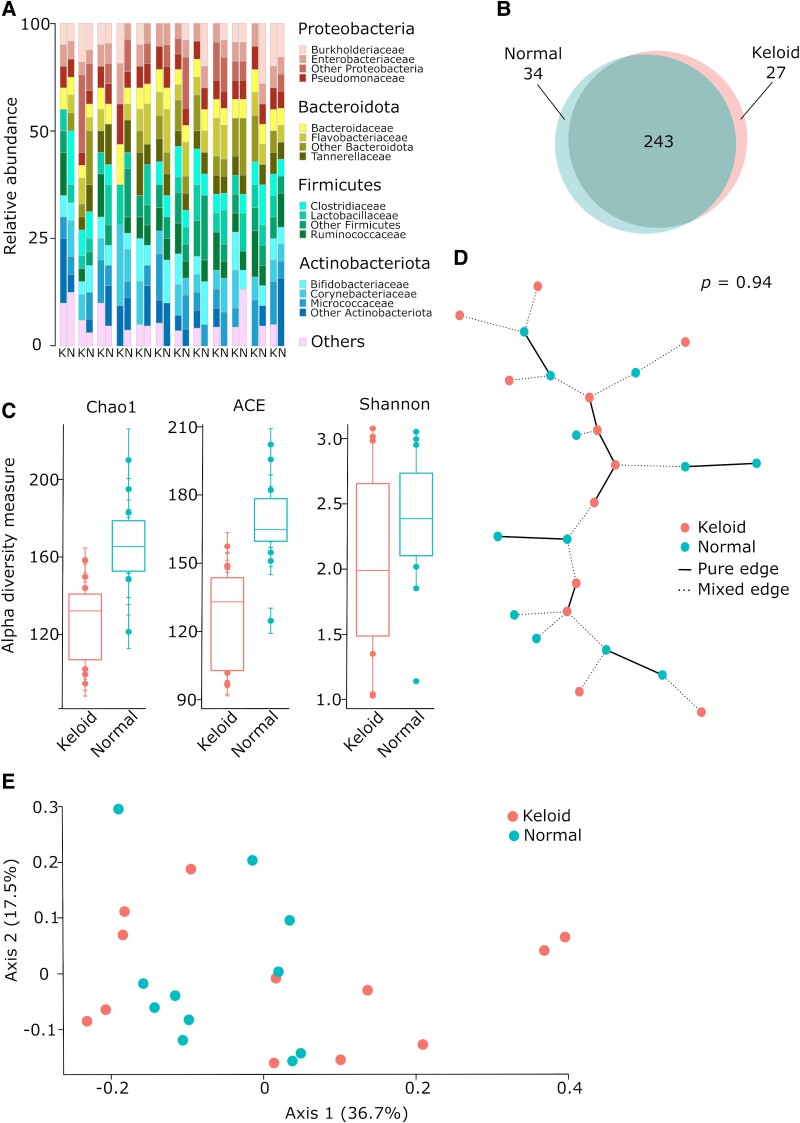
Composition of bacteria on skin surface of normal and keloid tissues. The swab samples were taken from keloids and normal skin (*n* = 12 in each group) and subjected to 16S rRNA sequencing and analysis. A) Composition of bacteria at the level of phylum and class. The graph shows the four most abundant phyla, while all other phyla are annotated as “Others.” In similar manner, each phylum also shows three the most abundant classes. Each column shows keloid (K) or normal (N) sample. The two adjacent columns from keloid and normal tissues represent data from the same patient. B) Venn diagram representing the number of taxa specific for each type of sample and shared between samples. C) The differences in alpha diversity measured by three different methods. D) Graph showing differences between composition of bacteria residing on the surface of keloids or healthy skin. E) Beta diversity analysis of swab assays between taken from healthy and pathological skin surface.

Possible bacterial dysbiosis in the epidermis, and later in the dermis, may result from a different composition of microbiome on epidermal tissues. As a scar tissue, keloids have a different composition of glands and hair follicles. The surface of skin in these areas shows different chemical composition and may support various bacteria that further infect the keloid scar. To test this point of view, we performed a swab test, collecting microbiota from the surface of keloid scar and adjacent healthy skin of patients. The material was further studied by 16S rRNA sequencing analysis. The microbiota composition on normal skin and keloids was similar but varied between specimens. The four most abundant phyla were *Proteobacteria*, *Bacteroidota*, *Firmicutes*, and *Actinobacteria* (Fig. 2A), typical groups identified in human skin. On both normal skin and keloids, the predominant genus was *Cutibacterium*, followed by *Staphylococcus*, *Corynebacterium*, and *Acinetobacter* (SI Appendix, Fig. S2A and B). Importantly, from over 300 identified amplicon sequence variants (ASVs), 80% were shared between healthy and keloid skin, 11% were specific for normal skin, and 9% for keloids (Fig. 2B). Alpha diversity values also confirmed this result. They are slightly lower in keloids, but not statistically different (Fig. 2C). Further, the graph permutation analysis shows that the distances among normal skin and keloids swab specimens are mixed with a high level of similarities (Fig. 2D). That is also confirmed by the small beta diversity of the samples (Fig. 2E). We did not find any significant results in the metagenomic functional analysis based on Kyoto Encyclopedia of Genes and Genomes (KEGG) pathways, except for four pathways with *P*-values <0.01 (SI Appendix, Fig. S2C). The composition of microbial communities is almost the same in keloids and normal skin on the tissue surface.

### Deep bacterial communities in healthy skin and keloid samples are different

**Fig. 3.**
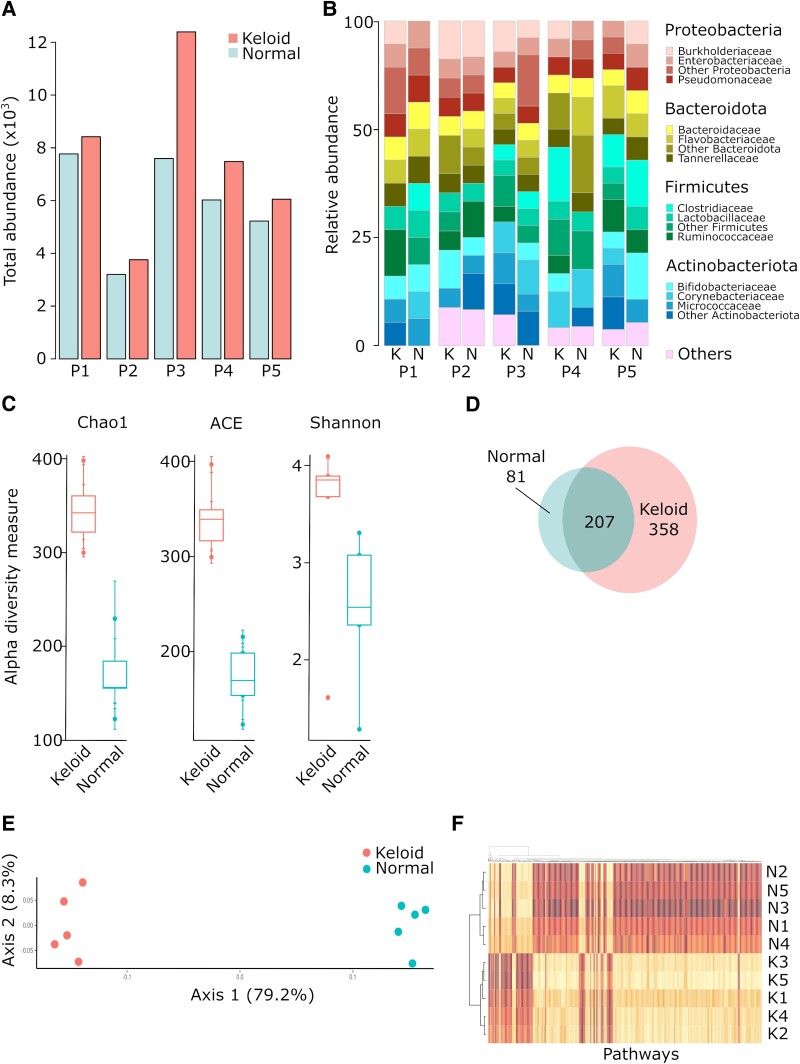
Bacterial community inside normal and pathological skin samples. Normal adjacent skin and central keloid tissues (*n* = 5 per group) were sampled from keloid-bearing patients and subjected to 16S rRNA analysis. A) Total abundance of bacteria in adjacent and pathological tissue. P1–P5, patient 1 to patient 5. B) Comparison of bacterial composition in keloid and adjacent healthy skin samples at phylum and class level. C) Comparison of alpha diversity between normal and keloid tissues. D) Venn diagram showing the specific and overlapped taxa number in normal and keloid microbial profiling. E) Analysis of beta diversity performed by PCA–wUnifrac method. F) Heatmap representing metagenomic analysis of microbiota colonizing normal and keloid samples. The data show functional pathways analyzed with tax4FUN method showing only the pathways with threshold *P*-value below 0.001. K, keloid; N, normal tissue; numbers mean patient identifier.

As shown before (Fig. 1), keloid samples compared to normal skin reveal a potentially higher number of bacteria. Thus, we tested bacterial composition also inside the keloid and healthy skin tissues. We collected samples from five keloid patients and compared the central part of the keloid with the marginal, healthy-looking adjacent skin. In all cases, the abundance of bacteria was higher in keloid tissue than in the healthy part, though the differences are variable (Fig. 3A). In contrast to the surface microbiota, the internal bacteria are different between keloid and normal tissues at the level of phylum and class (Fig. 3B). Similar to the surface bacteria, the predominant phyla are *Proteobacteria*, *Bacteroidota*, *Firmicutes*, and *Actinobacteria*. However, the alpha diversity of central keloid samples is significantly higher than normal skin (Fig. 3C), implying the dysfunction of the barrier. The surface composition of microbiota had twice as many ASVs as the swab tests. Both tissues share only 32% of ASVs, and 59% of detected taxons were keloid-specific (Fig. 3D, SI Appendix, Fig. S2D). In terms of beta diversity, the samples separate well (Fig. 3E) and the differences are statistically significant (SI Appendix, Fig. S2E). Finally, the metagenomic functional analysis shows 892 different metabolic functional pathways (*P* < 0.001) that are clearly divided between bacteria isolated from keloid and healthy skin and form specific clusters (Fig. 3F). The data indicate that keloid tissue has more bacteria, more diversity, and different compositions compared to the surface and normal skin microbiome.

### Bacteria-related pathways and mediators are present in keloid tissues

**Fig. 4.**
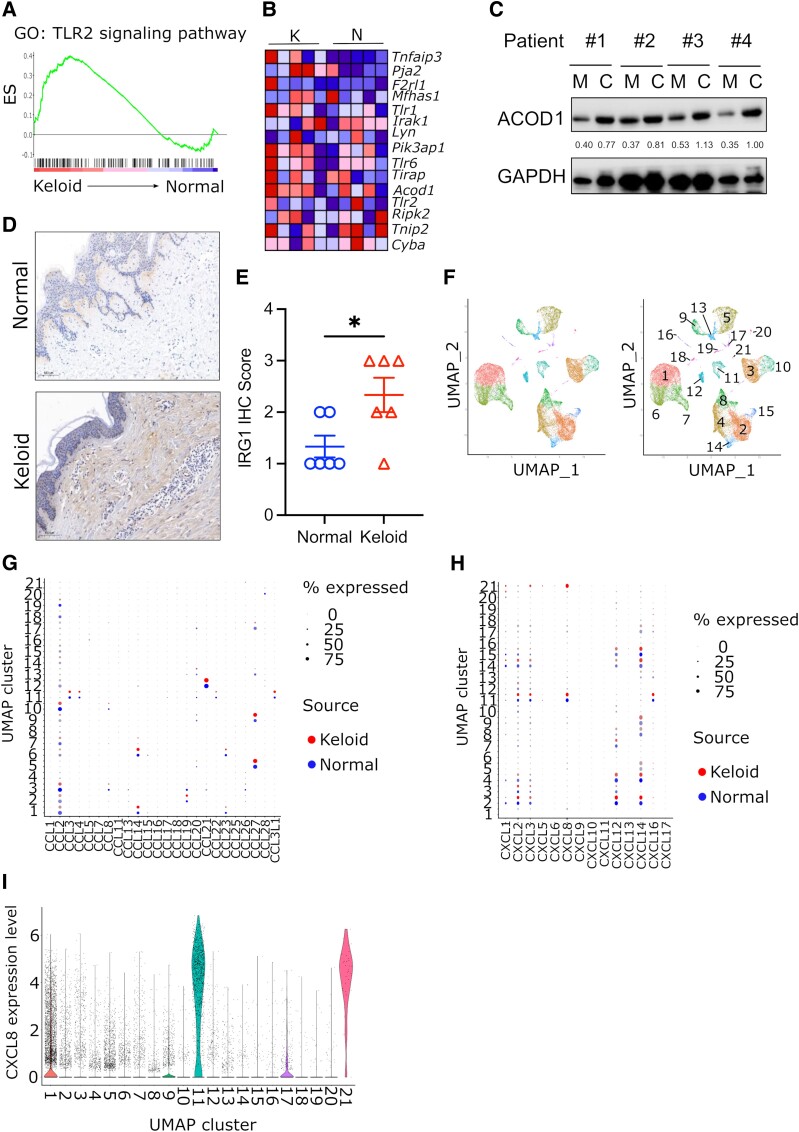
Activation of antibacterial pathways in keloids. Analysis of GeneChip data and single-cell RNA-Seq data from GSE7890 and GSE163973 datasets. The analysis was focused on cytokines and pathways associated with antibacterial response. We compared different clusters of fibroblasts isolated from healthy skin and keloid. A) Gene set enrichment analysis of TLR2 signaling pathway from dataset GSE7890. B) The main differentially expressed genes between keloid and normal skin from TLR pathway (GSE7890). C–E) Expression of ACOD1 protein in the keloid and adjacent normal tissues. Western blotting (C) was performed on protein isolated from four fresh patient samples. The numbers between lanes indicate the density ration of ACOD1 bands to relative GAPDH bands. For IHC staining, we used six archival paraffin-embedded samples. One representative image field of two fields obtained from one sample is shown on panel D), and scoring of the samples on panel E); *n* = 3 normal and three keloid samples, Student's t test, *P < 0.05. *F* clustering of cells from GSE163973 dataset. Expression of CCL (G) and CXCL (H) chemokines in each cellular cluster from dataset GSE163973. The dot size represents the percentage of cells expressing a given gene, while color discriminates keloid and normal tissues. I. Violin plots represent the expression of IL-8 in different clusters of cells from dataset GSE163973.

To further investigate the potential connection between microbiota and keloid tissues, we used publicly available GSE7890 (26) and GSE163973 (27) datasets deposited in Gene Expression Omnibus database. We found that in microarray data from keloids (GSE7890), proinflammatory pathways associated with bacterial activation, including TLR signaling, are induced compared with normal skin samples (Fig. 4A). Among the genes induced in keloids are *Tlr1*, *Tlr2*, *Tlr6*, and *Acod1* (Fig. 4B), commonly up-regulated by bacteria. Since *Acod1* gene expression is directly associated with the presence of bacteria, we further check it at the protein level. We noticed that ACOD1 expression is lower in the marginal part of keloids than in the central part of the keloid tissue at the whole protein level (Fig. 4C). Since skin tissue contains a lot of fat and extracellular collagen and the quantitation of protein for western blotting is not reliable, we further confirmed this result by IHC staining. It shows that keloid tissues display ACOD1 expression deep under the epidermis, while normal adjacent tissues display ACOD1 presence mostly close to the surface (Fig. 4D, E).

We further checked the dataset performed at the single-cell level (GSE163973). The cells from this set are divided into 21 clusters (Fig. 4F) with unique characteristics, including immune cells, fibroblasts, and epithelial and endothelial cells. The expression of TLRs in different clusters was not distinct between keloid and healthy tissues (SI Appendix, Fig. S3A). We also checked the expression of chemokines, which are frequently induced under inflammatory conditions. We did not find differential expression in most CCL (Fig. 4G) and CXCL (Fig. 4H) chemokines and their receptors (SI Appendix, Fig. S3B and C). However, a small cluster 21, which is present only in keloids, produces a high level of *IL-8* mRNA (Fig. 4I). The data show that keloid microenvironment may be linked to production of IL-8 induced by bacterial infection.

### Overproduction of IL-8 in keloids

**Fig. 5.**
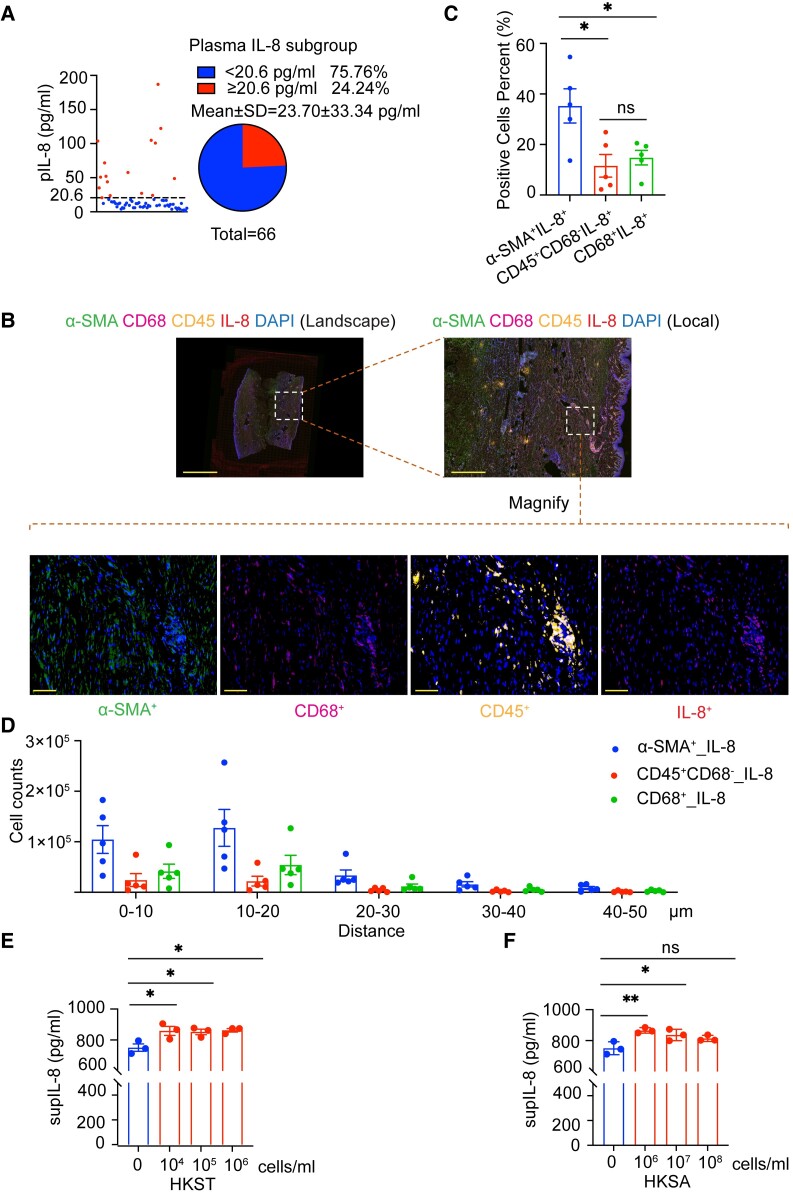
Fibroblasts are the feasible source of IL-8 in keloids. A) Percentage of patients in tested IL-8 chemokine subgroup in circulation. Reference values (RVs) are as follows: IL-8 = 0–20.60 pg/mL. The number of patients (*n* = 66) is indicated. B) Representative fluorescence image of samples from five patients showing the simultaneous detection of α-SMA, CD68, CD45, IL-8, and DAPI in human keloid. Scale bars, 100 μm. Magnification is 40×. C) Statistic analysis of indicated cell percent based on mIHC staining data from B). Mean ± SEM. ^ns^*P* > 0.05; *P < 0.05. Normality and log-normality tests followed by two-tailed unpaired Student's t test. D) Cell proximity analysis of indicated cells in the given distance based on mIHC staining data from B). Quantitative immunofluorescence was processed by HALO software. E) Dermal fibroblasts isolated from keloid tissues were treated with variable dosages of HKST for 24 h. IL-8 concentration in the supernatant was measured by ELISAs, *n* = 3 different keloid samples as a source of cells per subgroup. Normality and log-normality tests followed by one-way ANOVA were performed for the statistical analysis. Mean ± SEM. *P < 0.05. F) Keloid fibroblasts were treated with variable dosages of HKSA for 24 h. IL-8 concentration in the supernatant was measured by ELISAs, *n* = 3 per subgroup. Normality and log-normality tests followed by one-way ANOVA were performed for the statistical analysis. Mean ± SEM. ^ns^*p* > 0.05; *P < 0.05; **P < 0.01.

Next, we spot fibroblasts as potential IL-8 targets, as they express the receptors for IL-8 (28, 29). First, we tested blood from keloid patients. We screen several cytokines known as proinflammatory (Fig. 5A and SI Appendix, Fig. S4A) or antiinflammatory (SI Appendix, Fig. S4B) factors. Some of them, such as IL-1β or IL-5, are not detected in keloid patients, and others are present in range between 20 and 40% patients. IL-8 is one of the top three cytokines associated with keloids (Fig. 5A). Further, we studied the co-localization of IL-8 protein with tissue fibroblast and macrophage markers (α-SMA and CD68, respectively). Despite macrophages being potent IL-8 producers, we observed that α-SMA^+^ cells are the primary source of IL-8 in keloids (Fig. 5A–C). They are also the most closely located to IL-8 (Fig. 5D). Since we lack animal models of keloids, we used in vitro models. We treat the fibroblasts with heat-killed *Salmonella typhimurium* (HKST) or *Staphylococcus aureus* (HKSA), as examples of Gram-negative and Gram-positive bacterial strains. In both cases, the addition of bacteria induced the production of IL-8 (Fig. 5E and F). The data show that bacteria-activated fibroblasts produce IL-8 protein in the keloid environment.

### IL-8 modulates skin fibroblast behavior

**Fig. 6.**
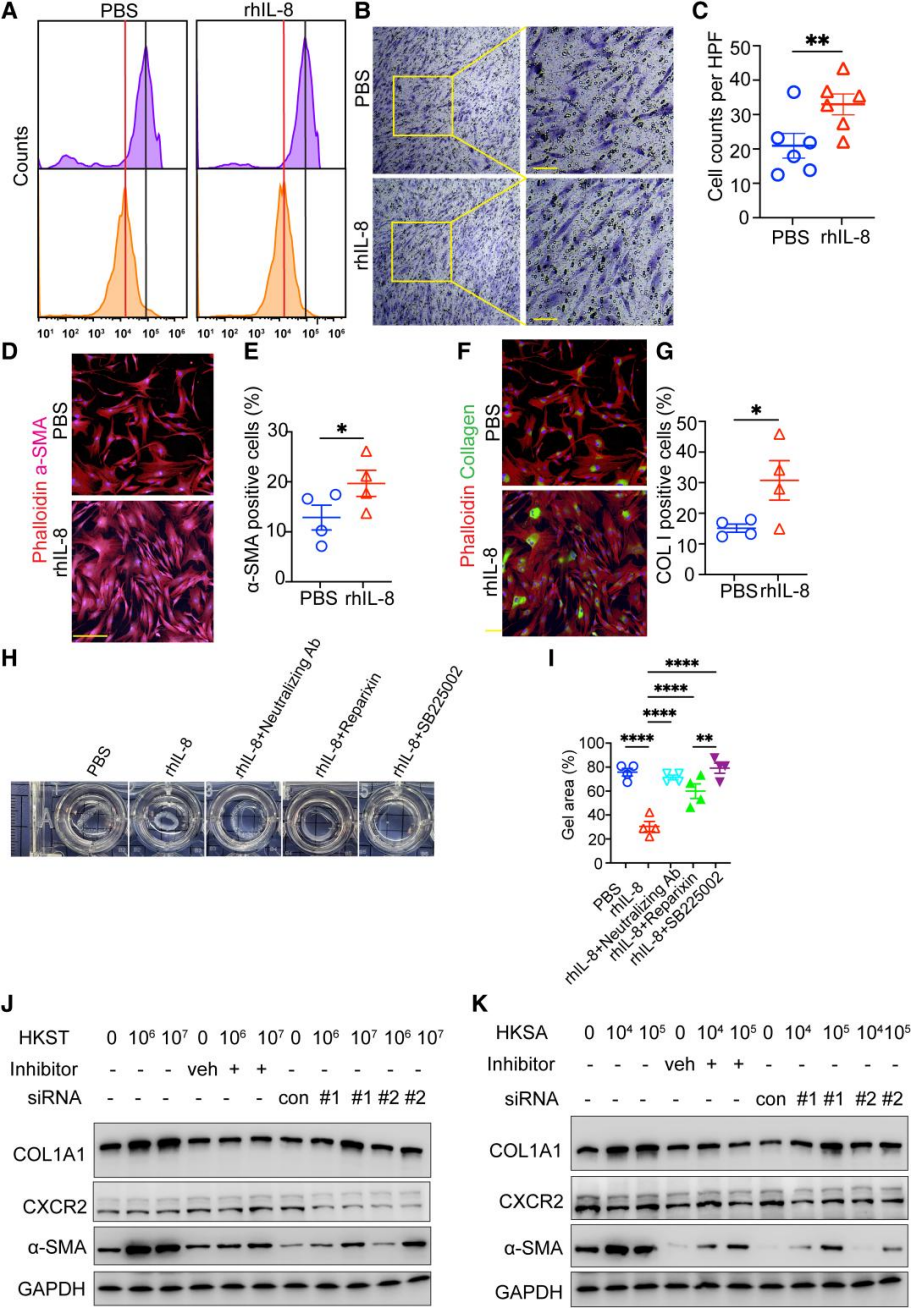
The role of IL-8 on phenotype and functionality of dermal fibroblast. A) FACS analysis on proliferation of CFSE-labeled dermal fibroblasts in response to vehicle (PBS) and rhIL-8 (50 ng/mL) (representative of two assays). B) Representative images of transwell assays on vehicle (PBS)- and IL-8 (50 ng/mL)-treated samples (*n* = 3 technical replicates, representative of two assays). C) Statistic analysis of cell number based on photographed images from B). Mean ± SEM. **P < 0.01. Normality and log-normality tests followed by two-tailed unpaired Student's t test were performed for the statistical analysis. D) Representative fluorescence images of independent samples from patients (*n* = 4) showing the detection of α-SMA. Phalloidin was used for reflecting cell morphology. Scale bars, 200 μm. E) Statistic analysis of α-SMA-positive cell percent based on photographed images from D). Mean ± SEM. *P < 0.05. Normality and log-normality tests followed by two-tailed unpaired Student's t test were performed for the statistical analysis. F) Representative fluorescence images of independent samples from patients (*n* = 4) showing the detection of collagen. Phalloidin was used for reflecting cell morphology. Scale bars, 200 μm. G) Statistic analysis of collagen positive cell percent based on photographed images from E). Mean ± SEM. *P < 0.05. Normality and log-normality tests followed by two-tailed unpaired Student's t test were performed for the statistical analysis. H) Gel contraction analysis of dermal fibroblasts with indicated treatments: rhIL-8 (50 ng/ml), rhIL with neutralizing antibody, rhIL-8 with CXCR1/2 inhibitor reparixin, and rhIL-2 with SB225002 antagonist of CXCR2 (*n* = 3 technical replicates, representative of two assays). I) Gel area (%) was calculated and analyzed based on the data from H). Mean ± SEM. **P < 0.01; ****P < 0.0001. Normality and log-normality tests followed by one-way ANOVA were performed for the statistical analysis. J, K) Dermal fibroblasts isolated from keloids were treated with variable dosages of HKST (panel J) or (panel K) for 24 h. Indicated proteins were detected by western blot. One of three western blots is shown. Keloid fibroblasts were treated with variable dosages of heat-killed bacteria in the presence of CXCR2 antagonist SB225002 or two different siRNAs. HPF, high-power field; Veh, vehicle for inhibitor; Con, siRNA control; #1, #2, two siRNAs.

To further determine the role of IL-8, we tested its effect on fibroblasts proliferation and migration. Cell counting assays and fluorescence-activated cell sorting (FACS) analysis on carboxyfluorescein succinimidyl ester (CFSE)-labeled primary fibroblasts upon IL-8 stimulation showed no significant effect on the proliferation (Fig. 6A and SI Appendix, Fig. S5A). Transwell assay showed increased fibroblast migration when treated with recombinant human IL-8 (rhIL-8) (Fig. 6B and C). Also, administration of rhIL-8 in scratch assay increased the ability of fibroblasts to migrate and seal the in vitro “wound” (SI Appendix, Fig. S4B and C). These data indicate that IL-8 increases the migration of skin fibroblasts.

Myofibroblasts, a distinctive subset of activated fibroblasts identified by α-SMA marker, have been implicated to be essential for extracellular matrix production (30, 31). We found the elevated expression of α-SMA in response to IL-8 treatment (Fig. 6D and E). Thus, we check the production of collagen, a key component of the extracellular matrix. Treatment with IL-8 promoted the production of collagen I (Fig. 6F and G). Moreover, a gel contraction assay showed enhanced contractility of IL-8-treated fibroblasts (SI Appendix, Fig. S6D and E). By contrast, neutralizing antibody of IL-8, CXCR1 inhibitor reparixin, or CXCR2 inhibitor SB225002 hindered the contractility of fibroblasts in the presence of IL-8 (Fig. 6H and I). We also showed microbial cues induced fibroblast–myofibroblast transformation, as evidenced by elevated expression of α-SMA and collagen I, which is reduced in the presence of CXCR2 inhibitor or CXCR2 small interfering RNA (siRNA) (Fig. 6J and K and SI Appendix, Fig. S6F and G). These data suggest that IL-8 signaling mediates skin fibroblast activation.

Next, we performed RNA-Seq to deeply explore the effect of IL-8 on skin fibroblasts. We observed that IL-8 treatment down-regulates the expression of ribosomal proteins handling protein synthesis (32, 33), including *RPL39*, *RPS21*, *RPS28*, and *RPS29* (SI Appendix, Fig. S6A–C). Expression of some components involved in mitochondrial respiratory chain, such as *MT-ND1*, *MT-ND2*, *MT-ND4*, *MT-CO3*, *MT-CYB*, *MT-ATP8* (34–36), were up-regulated (SI Appendix, Fig. S6A). They comprise essential bioenergetic pathways powering fibroblasts migration, to expedite healing (SI Appendix, Fig. S6B and C). It has been reported that *ZNF580* mediates H_2_O_2_-induced leukocyte chemotaxis by promoting the release of IL-8 (37), which implies a positive feedback loop augmenting the effect. We also noted that S100 calcium-binding protein A6 (*S100A6*), associated with proliferation, invasion, migration, and angiogenesis of cancer cells (38), was also down-regulated, showing similarities between tumor and wound healing events (SI Appendix, Fig. S6A).

Given the prominence of transforming growth factor β (TGF-β) in fibroblast activation, we finally sought to determine whether IL-8-mediated effects are like those of TGF-β. We compare the transcriptomes of fibroblasts in response to these factors. We found significant differences between them. Surprisingly, IL-8-treated samples showed decreased expression of genes involved in fibrotic events, such as *COL4A1*, *COL10A1* (39, 40), and *IL11* (41–44). However, it enhances canonical signaling driving fibrotic programs, as evidenced by up-regulated *TGFβR3* and *SMAD3* (45, 46) (SI Appendix, Fig. S6D–F). Thus, our findings suggest a profibrotic role for IL-8 upstream of TGF-β, which needs further exploration. Additionally, some biomarkers concerned with fibrosis, such as semaphorin 3A and phosphodiesterase-5A (47, 48), were also up-regulated in IL-8-treated samples compared with TGF-β-treated group (SI Appendix, Fig. S6D). These results collectively underscore the profibrotic effects of IL-8 on cutaneous fibroblasts, which are partially similar to the effects of TGF-β.

## Discussion

**Fig. 7.**
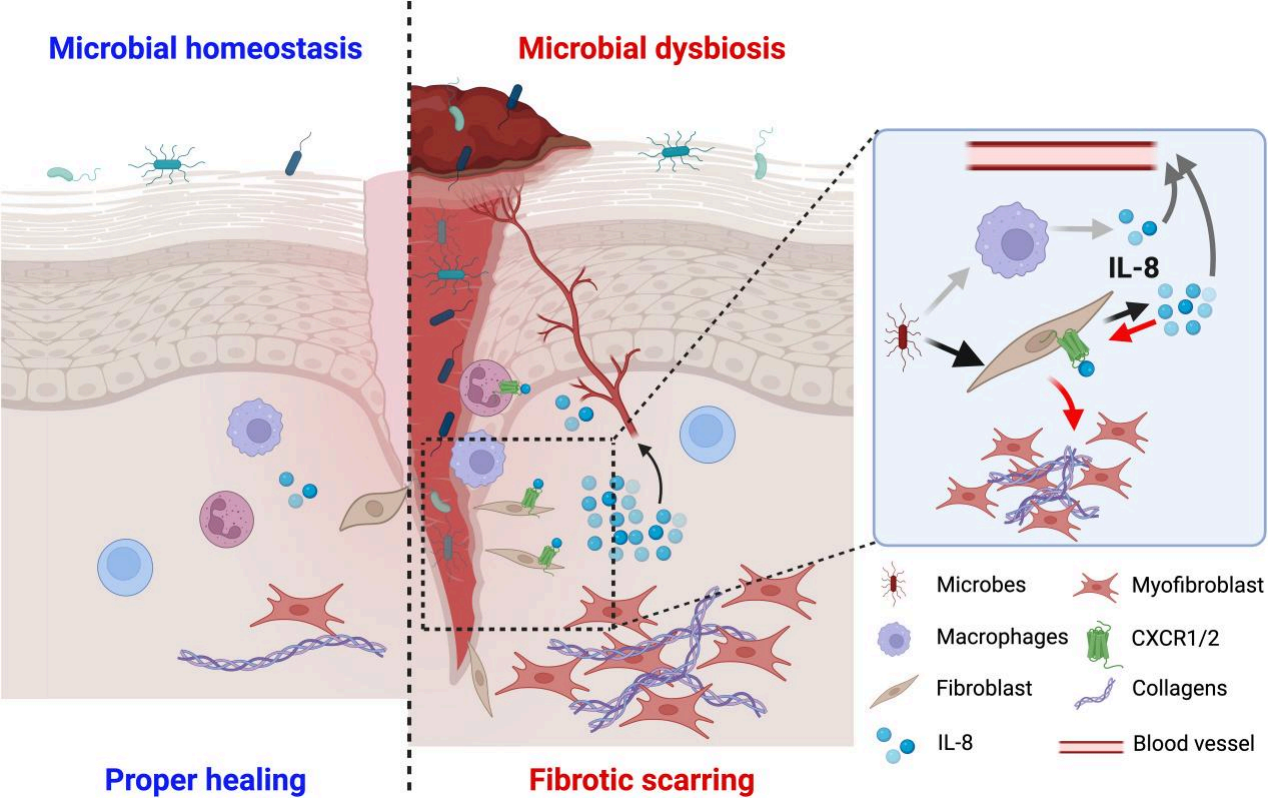
IL-8 and microbiota in keloids. Microbiota and IL-8 may play a role in the development or sustaining of keloids. Bacteria present in the keloid scar induce production of IL-8, which in turn can bind to CXCR1/CXCR2 receptors on fibroblasts and stimulate collagen deposition. IL-8 is also released to circulation, but in this case its further role needs to be determined. The figure was created with BioRender.

Keloids are specifically human skin disorder, lacking relevant animal models, and therefore difficult to study in terms of etiology and pathogenic mechanisms. While some genetic and environmental factors associated with keloid formation are identified (4, 6–11), none of them can be considered as a main explanation. It is highly probable that the formation of keloids is indeed a resultant of a few different factors. Current research on keloid pathology does not focus on the effects of skin microbiota, recognized as a critical component of skin biology. We asked whether bacteria live in keloid scars and what happens if they do. We found keloids contain bacteria and they induce IL-8 production, which may further support the fibrotic process in keloids (Fig. 7).

We know the interactions between skin and skin microbiota for decades. Bacteria are traditionally seen as pathogens that need to be eliminated. Examples include typical immune-related mechanisms, such as exfoliating the skin, producing antimicrobial peptides, and regulating the skin's pH. Bacteria, such as *Clostridium tetani* or *Clostridium perfringens* in wounds, can be deadly. However, microbiota can aid wound healing affecting the skin immune system (49–51). From this point of view, bacteria may be either beneficial or detrimental for the outcome of wound healing. For example, *Staphylococcus epidermis* induces CD8^+^ T-cell response and cytokine production, protecting against infection with pathogens (52) and supporting tissue repair (53). Staphylococcal LTAs also activate TLR3 signaling and reduces skin inflammation after wounding (54). However, skin injury is also a point of entry of microorganisms into deeper tissues (55), and bacteria play a critical role in pathological chronic wound healing, such as diabetic wounds (56). Together, those studies show that the healing process depends on the bacteria in the wound.

Keloids may be considered as wounds with not yet finished healing process. This agrees with the notion that keloid is a tumor-like disease, which shared features with wound healing (57, 58). The healing process is usually divided into a few steps: inflammation, proliferation, and remodeling (57, 59). After inflammation and proliferation steps, the freshly formed scar undergoes remodeling to finish scar tissue maturation. In keloids, the inflammation and proliferation steps continue, and microbiota colonizing them may play a role. As we show with a panel of cytokines, keloid patients show increased concentration of proinflammatory cytokines in the serum, including IL-12 and IL-8. Interestingly, IL-8 is also a well-known proangiogenic factor (60). Presence of IL-8 in both the serum and tissue of keloid patients suggests ongoing proliferation and angiogenesis. Bacteria also induce IL-8 production by activation of TLR signaling in a variety of cells in the body (61–63). Here, we show that IL-8 is also produced after stimulation with heat-killed bacteria by skin fibroblasts. Therefore, secretome of skin cells may be regulated by microbiota in keloids, affecting fibroblasts. Of note, we found a rare cell population that highly expresses IL-8 mRNA using the transcriptomic data (Fig. 4E and F). It remains undefined currently, but deserves further explorations.

From a practical point of view, this study shows a new potential direction for clinical treatment of keloids. Currently, the most common therapeutic regimens are surgical excision, intradermal corticosteroid injection, laser or radiation therapy, pressure dressings, and combinations of them (64–66). For example, only approximately 50% of keloids respond to corticosteroid treatment, whereas nearly 100% regrow after surgery used as the only therapy (65). Even with surgery and radiation therapy combined, 27% of our patients still had a relapse. None of these strategies targets microbiota, at least not directly. Antibiotics or phage treatments can improve outcomes for other therapies, such as surgery, by reducing bacterial burden. One of the problems may be the high variability of bacteria identified in keloids. However, high-throughput methods, such as 16S rRNA sequencing, are now more affordable for diagnostic purposes. In such a case, keloids can be treated with personalized medicine using antibiotics that match the patient's microbiome. Alternatively, identifying more molecular players between microbiota and keloids can lead to new target pathways. It is worthy to mention that growing evidence showed that IL-8 is such a molecule with prospective clinical value and deserves more attention (67–71).

This study has several limitations. First, it was known that keloid patients often have a history of folliculitis or minor lesions, such as scratches on the skin surface. Bacteria can enter the skin through wounds and cause fibrosis by producing IL-8. The limited body volume of samples makes it challenging to determine whether a particular bacterium is associated with keloids. This question requires further explorations with larger cohorts for a comprehensive understanding. Even more disturbing question is “hen-and-egg problem.” One symptom of keloid disease is itch; thus, the bacteria present in the keloid tissue may be introduced after keloid formation by the patient. In such case, the bacteria within the tissue may be the effect, not the cause of the disease. Keloid tissue conditions can cause different bacteria to grow on the surface versus the inside. Also, our results did not show other microorganisms, such as fungi, which has been showed that synergizing with bacteria in disease (72). This problem may be overcome by shotgun metagenomic analysis and internal transcriber spacer sequencing. Second, because of the human-specific character of keloids, we cannot use animal models and check the effect of bacteria in the in vivo setting. To this end, heat-killed bacteria are the way to stimulate in vitro, linking microbiota, fibroblasts, and IL-8. Third, though we focus on IL-8 cytokine herein, we cannot exclude other cytokines/inflammatory factors, perhaps even more important. Though we see the effects of IL-8 on fibroblasts, they are not as strong as expected. The experiments only focused on fibroblasts, without other vital cells such as macrophages and neutrophils, which could partially explain the effect. Ideally, experiments in the organoid environment containing multicellular modules concurrently would provide more comprehensive information, though it remains technically challenging to date. Therefore, we treat this study as a proof of the concept that microorganisms surrounding might be involved in the pathogenesis of fibrotic skin disease.

In conclusion, our research shows that bacteria colonize keloids, and microbiota composition differs from normal skin. These microorganisms possibly induce the secretion of soluble factors, including IL-8, that further affect shared pathological events of fibrotic disease evolution. Thus, our findings may stimulate further research on the bacteria and relevant pathways induced by their presence in keloids, which offer a promising avenue for translational practice.

## Materials and methods

### Human subjects

Keloids were diagnosed based on the history, anatomical location, clinical appearance, and pathology. Center and margin tissues were harvested during plastic surgery from patients confirmed by clinical evidence of keloid. Only stable keloids with no previous history of radiotherapy, chemotherapy, or intra-lesional steroids treatment prior to surgery were used in this research. Normal human upper eyelid skin samples were obtained from blepharoplasty of healthy donors. Keloid disease was confirmed by histopathologists. All samples were collected under ethical approval of the Medical and Ethics Committees of Renji Hospital, Shanghai Jiao Tong University. Each patient signed an informed consent before enrolling in this study.

### Cell lines and primary cultures

The adult human dermal fibroblast cell line (HDFα, FH1092, FuHeng Biology) was cultured in RPMI 1640 medium (11875093, Gibco) supplemented with 10% fetal bovine serum (FBS, 10099141C, Gibco) and 1% penicillin–streptomycin (10378016, Gibco). Cells were cultured at 37 °C, 5% CO_2_ conditions. The cells were authenticated by morphological examination and routinely tested for the absence of mycoplasma contamination. For isolation of primary fibroblasts, excised skin was immersed in saline containing antibiotics and antifungal agents (Anti-Anti, 15240062, Gibco) and transferred to the laboratory. After removal of the adipose tissue under the reticular dermis, samples were cut into ≤5-mm-diameter pieces and incubated with dispase II (4942078001, Roche) at 37 °C for 2 h. The epidermis was removed, and the dermis was minced and digested (37 °C, 1 h), following 4 °C overnight treatment with collagenase IV (17104019, Gibco). The cell suspension was filtered through a 70-μm cell strainer and centrifuged at 400*×g* for 10 min. The pellet was washed twice with PBS containing Anti-Anti at 400*×g* for 5 min. The cells were then resuspended in Dulbecco's Modified Eagle Medium (DMEM) (10569010, Gibco) with 10% FBS and 1% Anti-Anti for expanding.

### Immunohistochemistry

The tissues were fixed in 4% paraformaldehyde and paraffin-embedded. For further processing, 8-μm sections were used. For tissue morphology, we used hematoxylin and eosin (H&E) staining (60524, Yeasen). For IHC staining, we used antibodies against LPS core (HM6011, clone WN1222-5, HycultBiotech, 1:500), LTA (HM2048, clone 55, HycultBiotech, 1:200), or ACOD1 (201983-T08, Sinobiological, 1:2,000). Slides were scanned with an Aperio CS2 (Leica) automated slide scanner. The randomly selected visual fields were scored by the same observer following the standard pathohistological scoring method (0–4 score).

### Multiplex immunohistochemistry (mIHC) staining

Formalin-fixed and paraffin-embedded (FFPE) sections were evaluated by mIHC analysis following previously reported protocol (73) using antibodies for α-SMA (ab124964, Abcam, 1:500), IL-8 (MAB208, R&D Systems, 1:100), CD45 (2403794, Invitrogen, 1:200), and CD68 (76437, Cell Signaling Technology, 1:400). Cell nuclei were stained with 4,6-diamidino-2-phenylindole (DAPI, 62248, Thermo Scientific). Slides were scanned by a digital slide scanner, and fluorescent images were obtained and analyzed with the confocal microscope and software (Nikon) and ImageJ software (NIH, USA). For quantification of multispectral images, the slides were scanned using the Mantra System (PerkinElmer) and analyzed using HALO software (Indica Labs).

### Immunofluorescence staining

Immunofluorescence staining was performed by deparaffinization and rehydration, endogenous peroxidase quenching, acidic antigen retrieval, and blocking steps. Primary antibodies (anti-FAP, 66562, Cell Signaling Technology, 1:100; anti-COLIA1 BA0325, BOSTER, 1:200; anti-α-SMA, BM0002, BOSTER, 1:200) or phalloidin (Beyotime Technology, 1:100) were applied at 4 C overnight. Secondary antibodies (antimouse IgG1 AF647, 34113, Yeasen, 1:200; antirabbit IgG AF488, 34206, Yeasen, 1:200) were added for 1 h at room temperature (RT). Slides were mounted with DAPI Fluoromount-G (Yeasen, 36308) and scanned using the automated slide scanner.

### 16s rRNA FISH

FFPE tissues were used for previously reported 16S rRNA FISH protocol (74). Briefly, slides were deparaffinized, rehydrated, and incubated in 70% ethanol at 4°C for 3 h. Slides were then washed with 2× saline sodium citrate (SSC) buffer (AM9770, Invitrogen), which was used as a vehicle buffer in all further steps and washes. The slides were incubated with proteinase K (10 μg/mL, AM2546, Invitrogen) for 15 min. After two washes with 2×SSC and next two with 15% formamide, the slides were hybridized overnight at 30 °C with Cy5-labeled probes (specific probe: GCTGCCTCCCGTAGGAGT, nonspecific probe: CGACGGAGGGCATCCTCA, both at 1,680 nM) in 10% dextran (60316, Yeasen), 1 mg/mL *E. coli* tRNA, 0.02% bovine serum albumin (36106, Yeasen), 2 mM vanadyl-ribonucleoside (S1402S, New England Biolabs), and 15% formamide (60345, Yeasen). Sections were washed with 15% formamide at 30 °C. Cell nuclei were stained with DAPI in 15% formamide at 30°C. Slides were washed and mounted for scanning with the automated slide scanner.

### Microbiota collection, sequencing, and analysis

Surface or intra-tissue microbiota samples were collected from the pathological location or the normal lateral location of patients using a swab or punch biopsy (Catch-all Sample Collection Swab, Epicenter) moistened in Yeast Cell Lysis Buffer (MasterPure Yeast DNA Purification Kit; Epicenter). Samples were snap-frozen on dry ice, and DNA was isolated using the PureLink Genomic DNA Mini Kit (Invitrogen). Sequencing of 16S rRNA amplicons was conducted by Apexbio Co., Shanghai, China, using the Illumina NovaSeq platform with 150-bp paired-end V3 + V4 chemistry for the human samples.

Bioinformatics analysis was performed in R (75) v. 4.1.0. After initial trimming and filtering the raw data reads based on quality control, DADA2 algorithm (76) was used to get ASVs. After denoising and removal of chimeric sequences, the taxonomy was assigned based on SILVA v138 16S rRNA database (77) using DECIPHER package (78). Phylogenetic tree was constructed with phangorn (79). Sample data, taxonomy table, and phylogenetic tree were merged into phyloseq object and further analyzed using tools from phyloseq package (80). We filtered out the phyla that were present in <3 samples, agglomerated taxa at the genus level, and calculate relative abundances. Next, we calculated alpha and beta diversity. The analysis of minimum-spanning tree with Jaccard’s dissimilarity was performed with phyloseqGraphTest package (81). Krona charts were prepared using KronaTools (82). Functional pathways associated with microbiota were analyzed using tax4Fun from themetagenomics package (83).

### Nontargeted metabolomics

Nontargeted metabolomic analysis was performed using an Agilent gas chromatography–mass spectrometer (7890A/5975C GC-MS System, Agilent, CA, USA) and conducted by Apexbio Co.

### Three-dimensional collagen gels

Primary human dermal fibroblasts suspended in serum-free medium were mixed with 4 mg/mL of neutralized rat tail type I collagen at a ratio of 3:1. The mixture was seeded in 12-well plates at a cell density at 1 × 10^5^/mL. Gels were coagulated at 37 °C for 1 h. The edge of the gel was detached from the well wall to allow contraction, and 1 mL culture medium (with/without 100 ng/mL rhIL-8) was added. 48 h later, the gel area was measured on pictures using ImageJ.

### Flow cytometry

Keloid fibroblasts labeled with CFSE (C1031, Beyotime Biotechnology) were harvested and suspended in 500 μL of FACS buffer and acquired by flow cytometry (BD FACSVerse). The data were analyzed using FlowJo software.

### Dermal fibroblasts scratch assay

Confluent HDFα cells were pretreated with vehicle or 50 ng/mL of rhIL-8 for 12 h before scratch assay. Scratches were created by manually scraping the cell monolayer with a 200-μL pipette tip. Cells were constantly treated with rhIL-8 (50 ng/mL). The scratch area was imaged using Nikon Ti2-U microscope and quantified using ImageJ software.

### Transwell assays

HDFα cells were evaluated for their ability to migrate a wound site through a transwell assay. Cultured cells upon vehicle or IL-8 treatment were seeded in the upper chamber of the transwell plates and allowed migrating for 24 h. Then, those cells transferred into lower chamber were fixed in PFA for 30 min at RT, stained with 0.4% trypan blue for 30 min, washed 3 × 10 min with PBS, then dried at RT, and photographed under a 10× microscope (Ti2-U, Nikon), and cell numbers in the field were calculated manually by at least two colleagues (*n* = 6).

### RNA isolation and sequencing

RNA was extracted using RNeasy Mini Kit (Qiagen). RNA purity and concentration measurement, preparation of RNA library, and transcriptome sequencing were conducted by Tiangen Co. Genes with adjusted *P* value <0.05 and log2 (FoldChange) > 0 were considered as differentially expressed. Heatmap was generated using R software.

### Immunoblotting

Proteins were extracted with radio-immunoprecipitation (RIPA) lysis buffer (P0013C, Beyotime Biotechnology), and concentrations were measured by the Bradford assay kit (P00006C, Beyotime Biotechnology). Protein extracts were separated by sodium dodecyl sulfate–polyacrylamide gel electrophoresis (SDS–PAGE), transferred to nitrocellulose membranes (Millipore), and blocked with 5% nonfat dry milk. The membranes were incubated with primary antibodies against α-SMA (19245, Cell Signaling Technologies, 1:1,000), collagen (Col1A1) (BA0325, BOSTER, 1:2,000), glyceraldehyde-3-phosphate dehydrogenase (GAPDH) (30201, Yeasen, 1:2,000), or cis-aconitate dehydrogenase 1 (ACOD1) (ab222411, Abcam, 1:1,000) in Tris-buffered saline–Tween (TBST, 10 mM Tris–HCl, pH 7.5, 150 mM NaCl, 0.05% Tween 20) overnight at 4°C. After washing with TBST, the membranes were incubated with horseradish peroxidase-conjugated antimouse or antirabbit secondary antibodies (Epizyme Technologies) for 1 h at RT and washed with TBST. The membranes were incubated with enhanced chemiluminescence (ECL) reagents (Tanon) and visualized using a Bio-Rad ChemiDoc Imaging System (Bio-Rad).

### Small interfering RNAs

siRNAs for CXCR2 (Gene ID: 3579) were designed and synthesized by Tsingke Biotech (Beijing) to target two distinct sequences (#1 sense 5′–3′: GCGCUACUUGGUCAAAUUC(dT)(dT) and #2 sense 5′–3′: GCUCCGUCACUGAUGUCUA(dT)(dT)). A scrambled siRNA served as control. Cells were transfected with the 50 nM siRNAs using the Lipofectamine RNAiMAX (Invitrogen) according to the manufacturer's instructions. The extracts of RNAi and other groups were collected 96 h post-transfection and subjected to western blot analysis with the respective antibodies.

### Pathogens

HKST (tlrl-hkst2) and HKSA (, tlrl-hksa) were purchased from InvivoGen.

### Enzyme-linked immunosorbent assay

HDFα cells were treated with diverse dosages of HKST or HKSA for 24 h. Secreted IL-8 was measured using antihuman IL-8 ELISA kit (abs510004, Absin) according to the manufacturer's instructions.

### Cytometric bead array cytokine assay

Patients' blood was collected in ethylenediaminetetraacetic acid (EDTA)-coated tubes. The plasma cytokine level was quantitated using cytometric bead array kit (P110100403, JiangXi Cellgene) on FASCantoII (BD). Analysis was handled according to the manufacturer's instruction. Cytokine concentrations were analyzed by FCAP Array v3.0.1 software and BD FACSDiva software.

### Statistical analysis

The results represent means ± SEM. Data were analyzed using Prism 9.0 (GraphPad Software, San Diego, CA, USA). Statistical significance was determined by an unpaired two-tailed Student's t test when comparing two groups or one-way ANOVA when comparing more than two groups. Differences with *P*-values <0.05 were considered significant.

### External data

To identify cytokines or chemokines specific for keloid, we obtained publicly available Affymetrix microarray (GSE7890) (26) and single-cell RNA-Seq (GSE163973) (27) datasets from Gene Expression Omnibus database. Microarray data were analyzed using GSEA2 software (84), while scRNA-Seq results were analyzed with tools provided by Seurat (85) R package.

## Supplementary Material

pgae273_Supplementary_Data

## Data Availability

All data generated during this study are included in the manuscript and SI Appendix. The raw and processed data of 16S rRNA sequencing and RNA sequencing are submitted and available via DataDryad database, https://doi.org/10.5061/dryad.d51c5b0bt.

## Preprints

An earlier version of this manuscript was posted on bioRxiv: doi: https://doi.org/10.1101/2023.09.19.558395.
