# Supplementary material for: Commensal microbiome dysbiosis elicits interleukin-8 signaling to drive fibrotic skin disease
Source: PNAS Nexus. 2024 Jul 30;3(7):pgae273. doi: 10.1093/pnasnexus/pgae273 (PMC11287872; doi:10.1093/pnasnexus/pgae273)
Supplement: pgae273_Supplementary_Data [file pgae273_supplementary_data.docx]

**Supporting Information for**

**Commensal microbiome dysbiosis elicits IL-8 signaling to drive** **fibrotic skin disease**

Wenyu Zhang^1,#^, Qili Peng^2,#^, Xian Huang^1^, Qing Huang^1^, Zhiliang Zhang^2,3^, Fuli Li^1^, Naisheng Zheng^1^, Binsheng Shi^4^, Zhihong Fan^2,3^, Tomasz Maj^1,*^, Rui Chen^1,2,*^

* Corresponding authors:

Rui Chen: [dr.chen@shsmu.edu.cn](mailto:dr.chen@shsmu.edu.cn)

Tomasz Maj: [tomasz_maj@sjtu.edu.cn](mailto:tomasz_maj@sjtu.edu.cn)

**This PDF file includes:**

Appendix of supporting Figures1 to 6 and supplementary table 1.


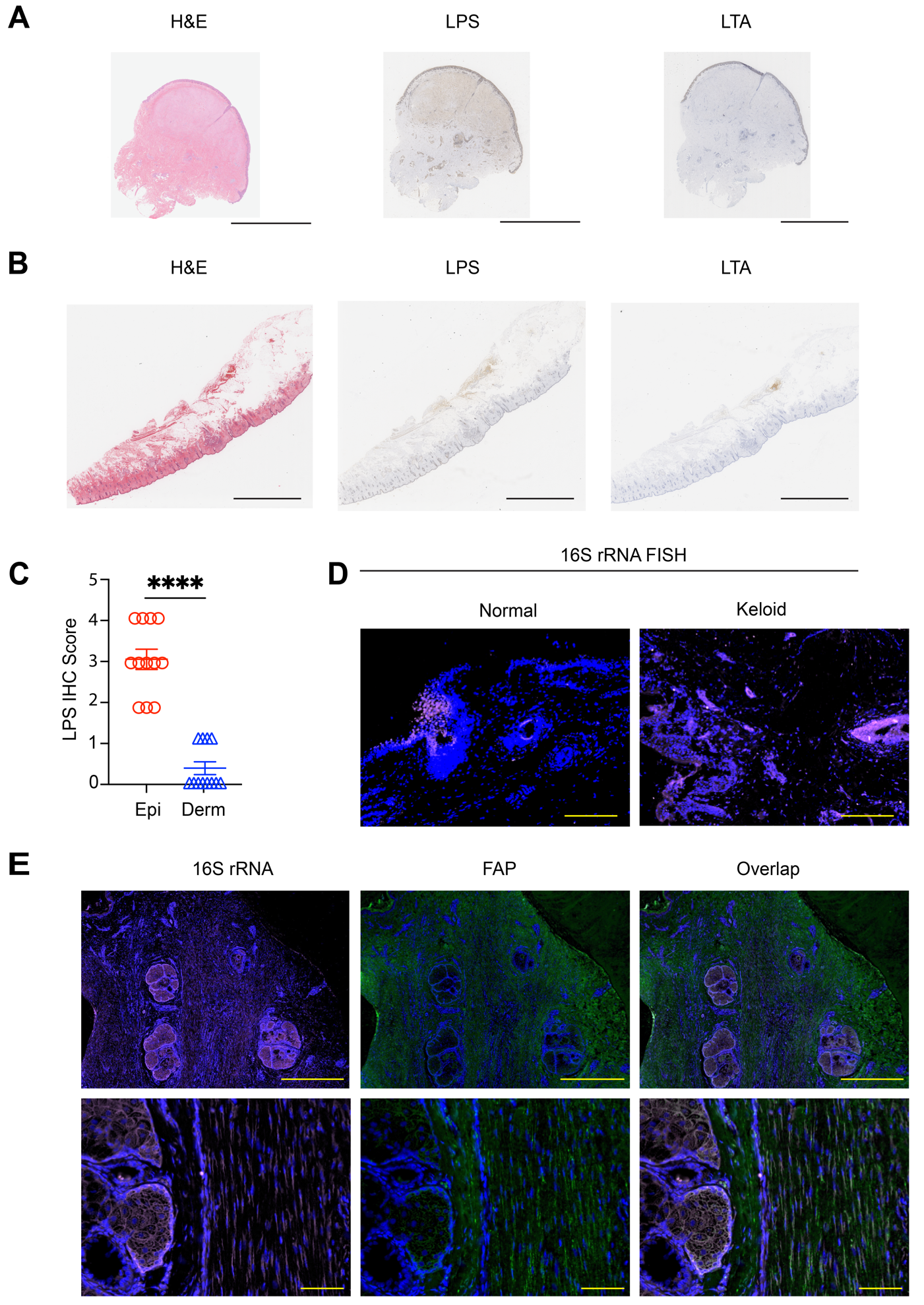


Fig. S1. Colonization of keloid by bacteria.

(**A**) Global visualization of H&E and bacterial LPS, LTA in keloid tissues. n=12. Scale bars, 5000 μm. (**B**) Global visualization of H&E and bacterial LPS, LTA in normal tissues (upper eyelid skinn). n=2. Scale bars, 5000 μm. (**C**) Comparison of IHC scores of LPS and LTA staining in two layers of keloid skin (Epi and Derm, n=12). Mean ± SEM. ****p < 0.0001. Normality and Lognormality Tests followed by two-tailed paired Student’s t test. (**D**) Normal (upper eyelid skinn) and keloid tissues were subjected to 16S rRNA FISH, with the pan-bacteria EUB338 probe. Scale bars, 200 μm. (**E**) 16S rRNA FISH and FAP-IF staining on consecutive slides for representative keloid tissues are presented. n=6. Scale bars represent 1000 µm for low magnification images (top row) and 100 µm for high magnification images (bottom row).


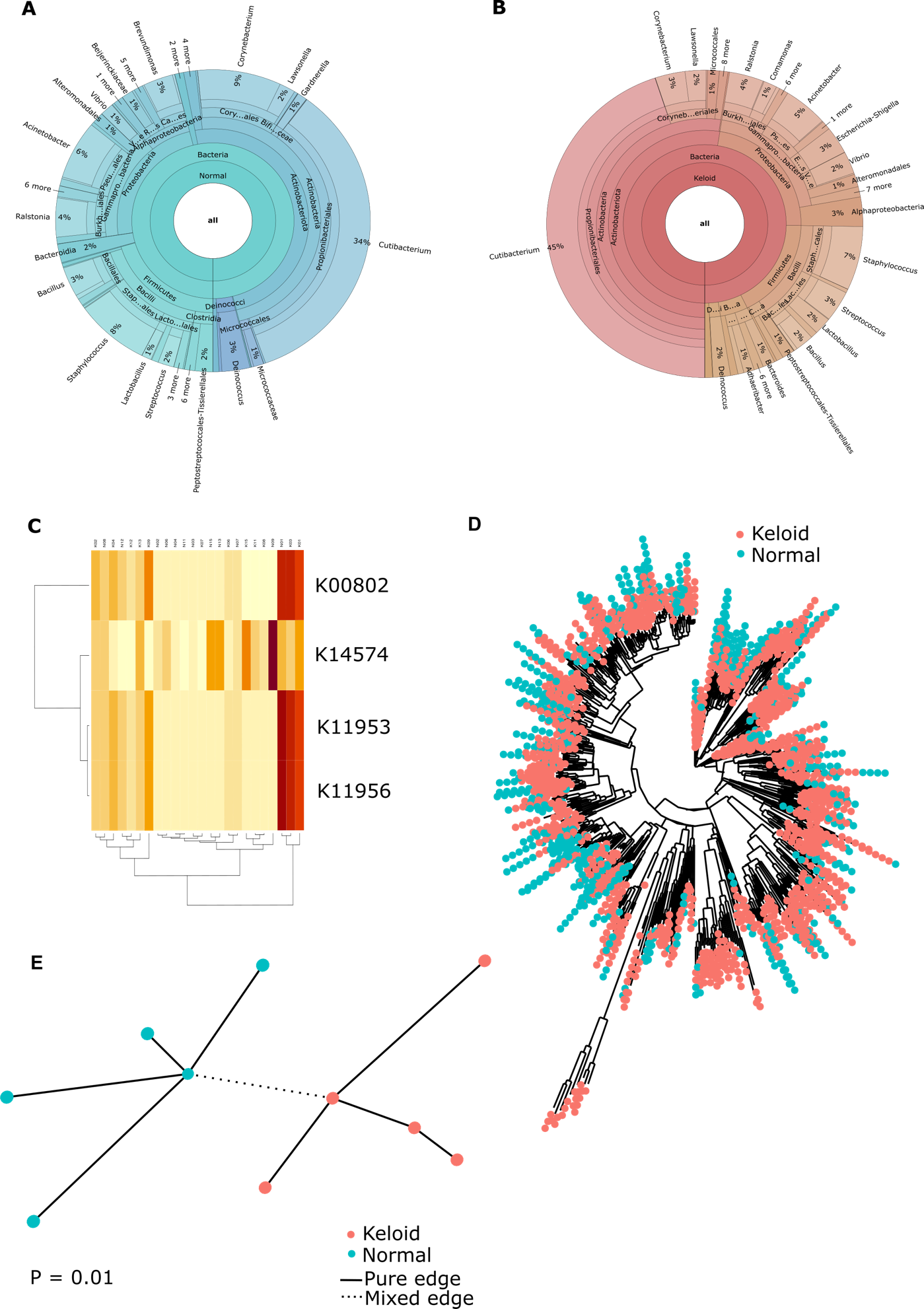


Fig. S2. Signature of bacteria in keloid.

(**A, B**) KRONA summary of bacterial communities analyzed by swab test on the surface of normal skin (**A**) and keloid scar (**B**). (**C**) Functional analysis of pathways distinctive for keloid and normal skin. The samples are shown in columns, where names beginning with N were aquired from normal skin, while samples with names beginning with K were collected from keloids. (**D**) Phylogenetic tree of microbiota detected in the keloid and normal tissues. Each dot at the tip of tree means the presence of a given AVS in the collected material. E.g., three green dots and two red dots means that the AVS was present in three out of five normal skin samples, and two out of five keloid samples. (**E**) Permution test and tested network of bacterial communities in keloid and normal tissue samples.


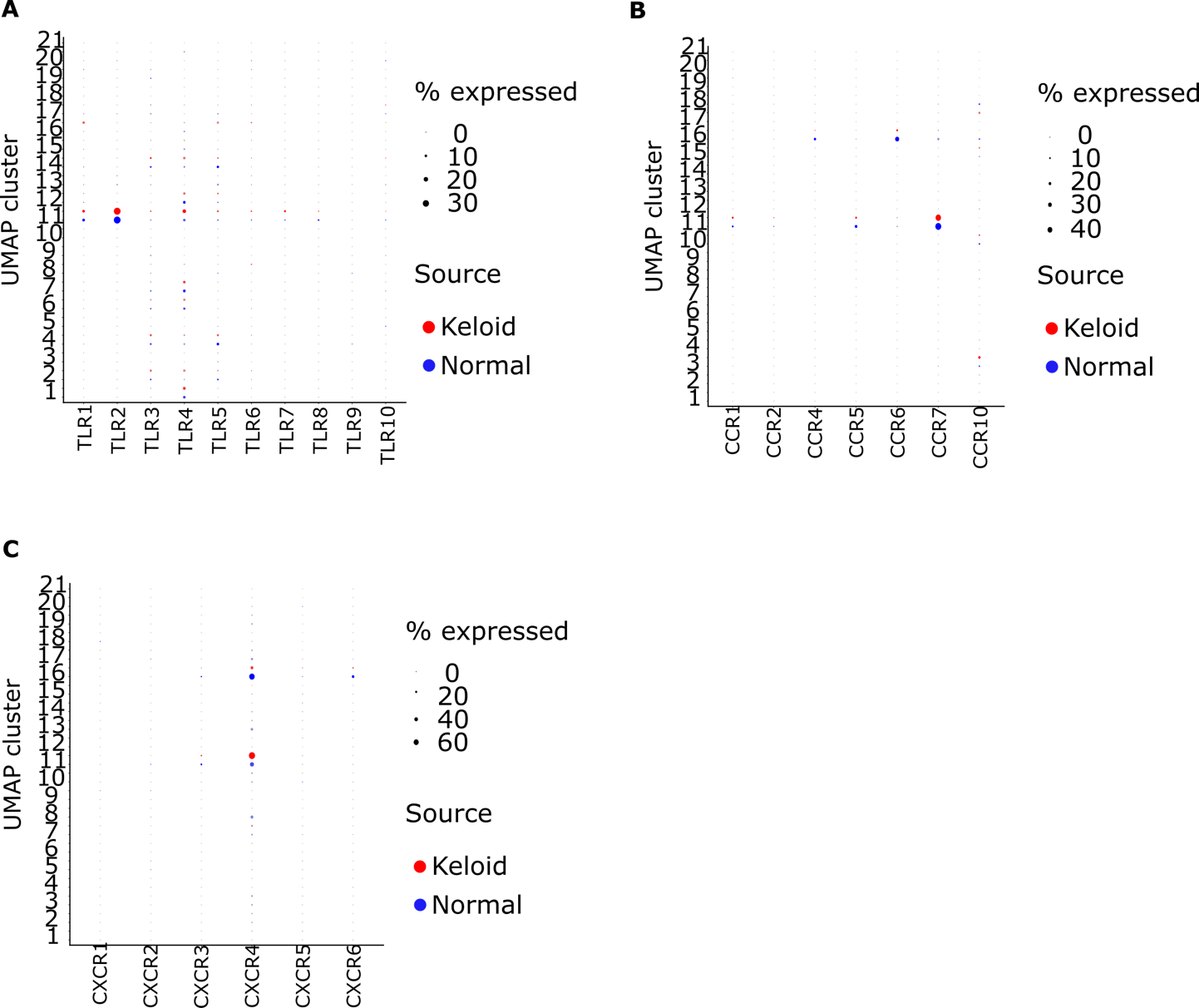


**Fig. S3. Receptors signatures in identified subsets**

The expression of TLR receptors (**A**), CCL chemokines receptors (**B**), and CXCL chemokines receptors (**C**) in cellular clusters identified in dataset GSE163973.


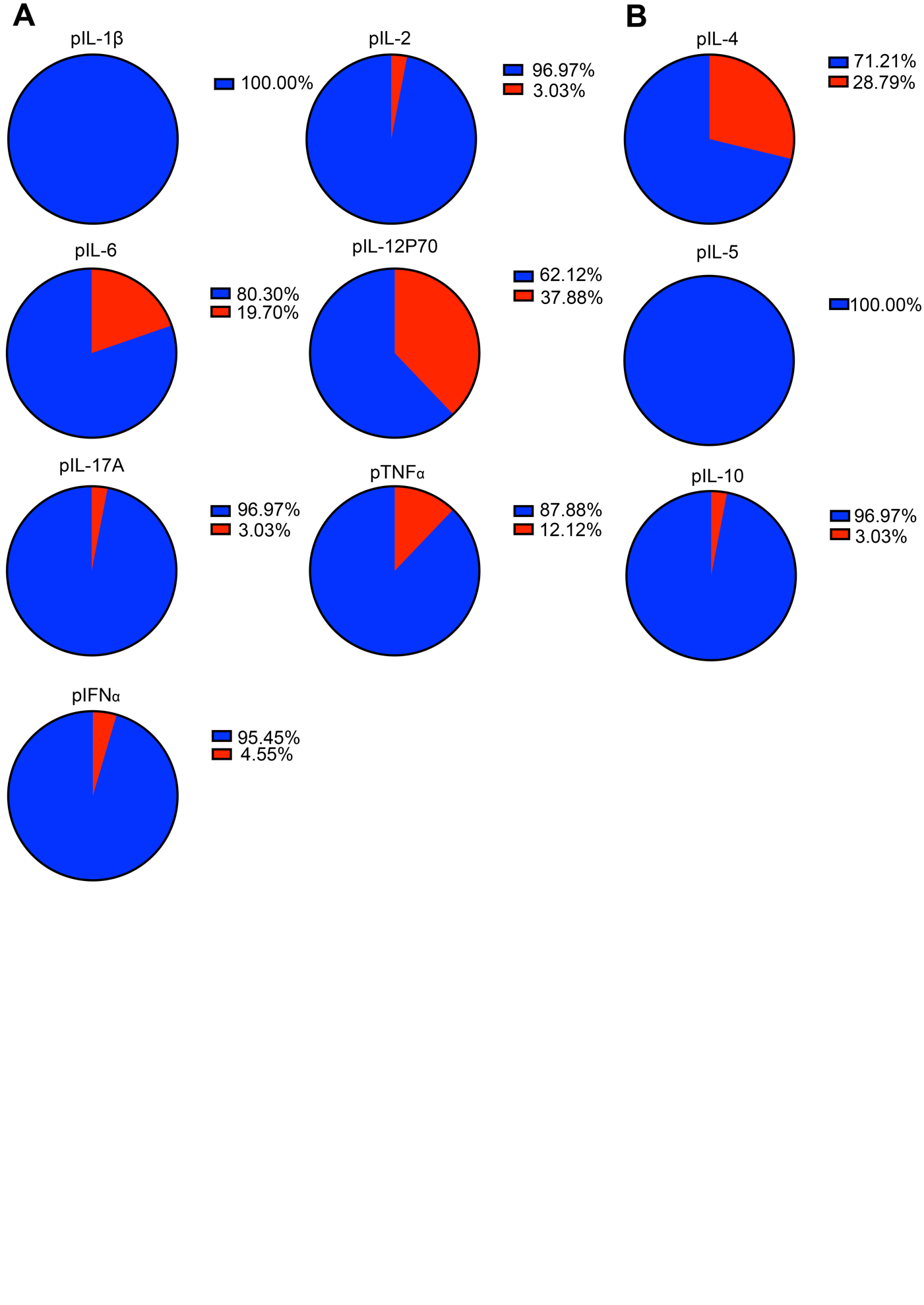


Fig. S4. Clinical tests for relevant cytokines from patients’ blood samples.

(**A**) Percentage of patients in tested inflammatory cytokine subgroup in circulation. Reference values（RVs） are as below: IL-1β=0~12.40 pg/ml, IL-2=0~5.71 pg/ml, IL-6=0~5.30 pg/ml, IL-12P70=0~3.40 pg/ml, IL-17A=0~20.60 pg/ml, TNFα=0~4.60 pg/ml, IFNα=0~7.42 pg/ml. (**B**) Percentage of patients in tested anti-inflammatory cytokine subgroup in circulation. RVs are as below: IL-4=0~3.00 pg/ml, IL-5=0~3.10 pg/ml, IL-10=0~4.91 pg/ml. Color in red indicates abnormal（above RVs）section. Color in blue indicates normal（within RVs）section. Total samples = 66.


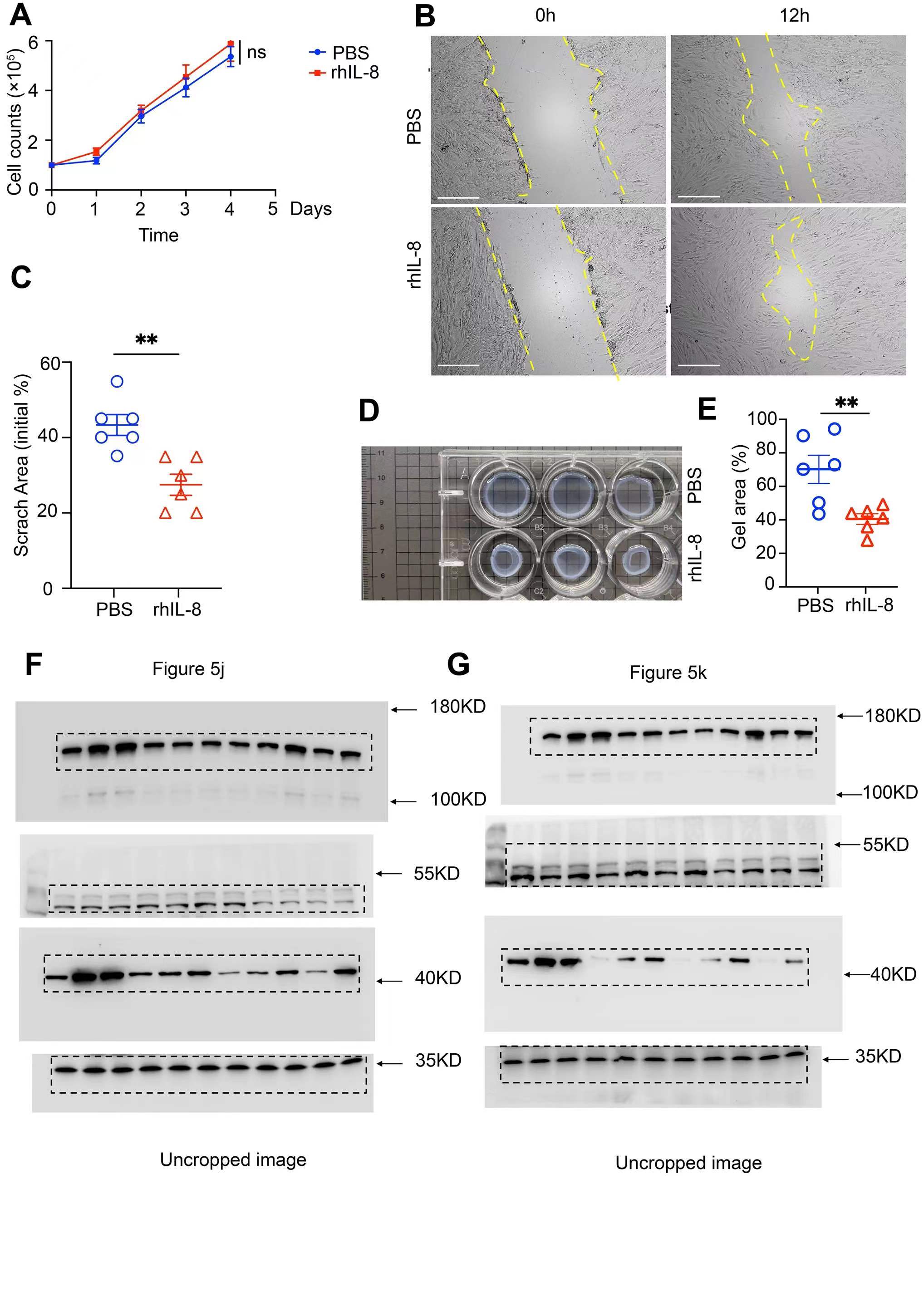


Fig. S5. IL-8 mediates the changes in skin fibroblast behavior.

(**A**) Cell Count Assays were executed by using automatic counter under indicated treatments: vehicle (PBS) and rhIL-8 (50 ng/ml). Data were collected at indicated time course and collectively showed as the growth curve (n=3). (**B**) ﻿Representative images showing rh﻿IL-8 (50ng/ml) stimulation promotes the migration of primary human fibroblasts. Yellow dashed line marks scratch wound edges (n = 3 technical replicates, representative of two assays). (**C**) Scratched areas at 12h post wound are quantified as the percentage relative to the initial area at 0 hours. Scale bars, 100 mm. Mean ± SEM. **p < 0.01. Normality and lognormality tests followed by two-tailed unpaired Student’s t test. (**D**) Gel contraction analysis of dermal fibroblasts treated with rhIL-8 (50 ng/ml) . n = 4. (**E**) Gel area (%) were calculated and analyzed based on the data from (**D**). Mean ± SEM. **p < 0.01Normality and lognormality tests followed by Student’s t-test. (**F**) and (**G**) Uncropped blots related to Figure 6J and Figure 6K are shown, respectively.


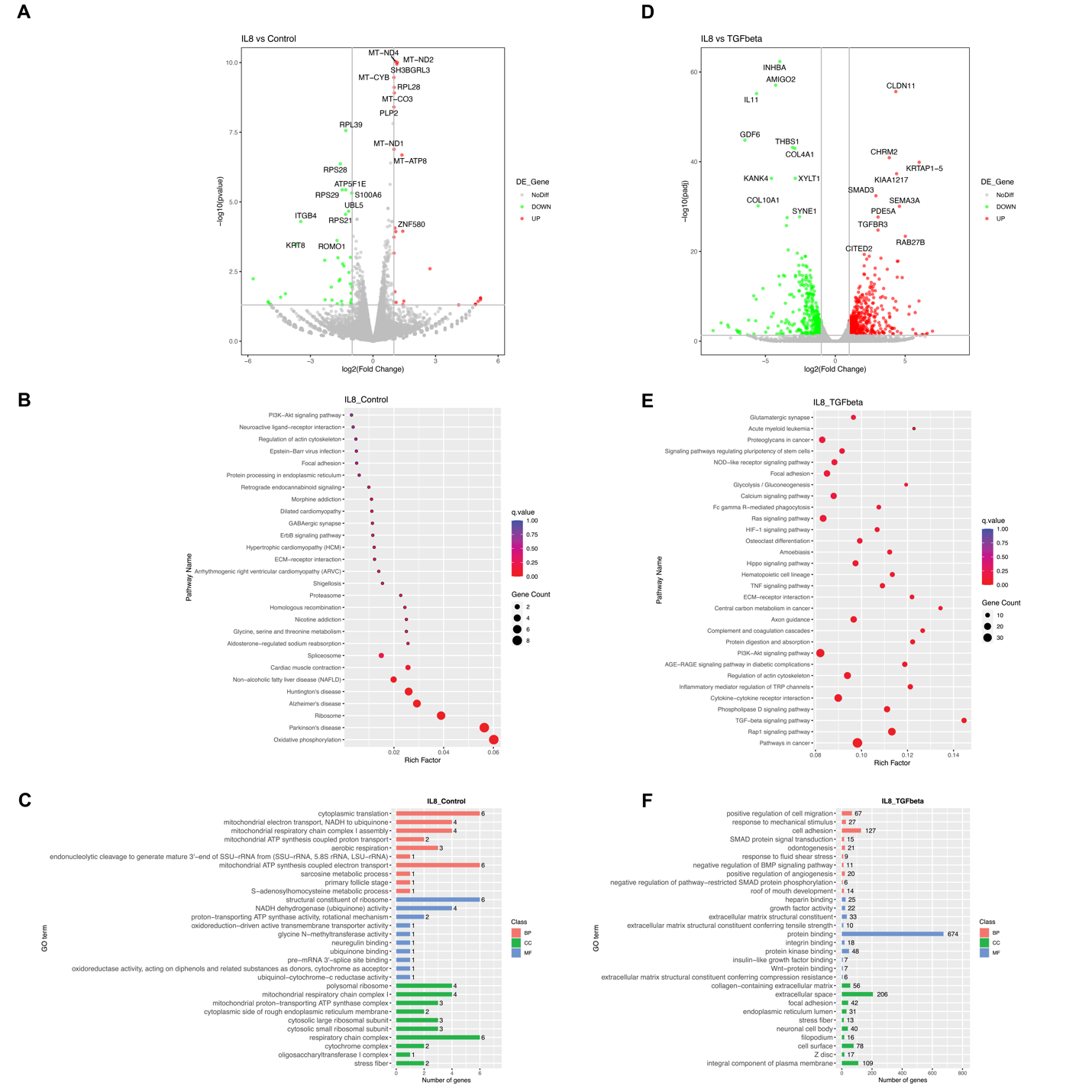


Fig. S6. Transcriptional shifts of the fibroblast upon IL-8 stimulation.

(**A**) Differential expression analysis of response in freshly isolated dermal fibroblasts upon vehicle and rhIL-8 (50ng/ml). Significant upregulated genes (red) and downregulated genes (green) are shown, Top 10 genes are marked as indicated. (**B**) Gene Set Enrichment Analysis (GSEA) for genes from (**A**). (**C**) Gene Ontology（GO）analysis for genes from (**A**). (**D**) Differential expression analysis of response in freshly isolated dermal fibroblasts upon IL-8(50ng/ml) and TGFβ (10ng/ml). Significant upregulated genes (red) and downregulated genes (green) are shown, Top 10 genes are marked as indicated. (**E**) Gene Set Enrichment Analysis (GSEA) for genes from (**D**). (**F**) Gene Ontology（Go）analysis for genes from (**D**). Two independent experiments were executed on primary dermal fibroblast isolated from keloid samples of 2 patients.

**Supplementary Table 1.** **Patient demographics and characteristics.**

| Metric | Value |
| --- | --- |
| Sex |  |
| Male | 28 (40%) |
| Female | 42 (60%) |
| Age |  |
| Mean±SD | 40.91±15.79 |
| Median | 36 |
| Site of lesion |  |
| Ear | 13 (18.6%) |
| Chest | 33 (47.1%) |
| Belly | 18 (25.7%) |
| Scapular | 6 (8.6%) |
| Lesion size |  |
| Small（<2cm^2^） | 6 (8.6%) |
| Medium(2-5cm^2^) | 22 (31.4%) |
| Large(>5cm^2^) | 42 (60%) |
| Mean±SD | 17.51±25.11 |
| Duration of the year |  |
| >1years and <2 years | 19 (27.1%) |
| ≥2 years | 51 (72.8%) |
